# Effect of Spirulina on the Rumen Microbiota and Serum Biochemical Parameters of Lambs

**DOI:** 10.3390/microorganisms12122473

**Published:** 2024-12-01

**Authors:** Zhijun Wang, Xiangdong Liu, Muqier Zhao, Weiqin Ma, Yuxuan Wang, Yushan Jia, Gentu Ge

**Affiliations:** 1 Key Laboratory of Forage Cultivation, Processing and Highly Efficient Utilization of Ministry of Agriculture and Rural Affairs, College of Grassland Science, Inner Mongolia Agricultural University, Hohhot 010019, China; zhijunwang321@126.com (Z.W.); 18847788933@163.com (X.L.); zhaomuqier@163.com (M.Z.); maweiqin0108@126.com (W.M.); xuanjqka@gmail.com (Y.W.); jys_nm@sina.com (Y.J.); 2 Key Laboratory of Grassland Resources, Ministry of Education, College of Grassland Science, Inner Mongolia Agricultural University, Hohhot 010019, China; 3 College of Grassland Science, Inner Mongolia Agricultural University, Hohhot 010019, China

**Keywords:** Hu sheep, Spirulina, diet, rumen microbiome, serum parameters

## Abstract

Spirulina (*Arthrospira platensis*) is rich in a variety of fermentable fibers and prebiotics, which can promote the proliferation of beneficial flora in the intestinal tract of ruminants and optimize the balance of microorganisms in the rumen. The aim of this study was to evaluate whether dietary supplementation with Spirulina has a beneficial effect on the rumen microbial community and serum indices in lambs. For this purpose, 36 lambs with a mean weight of 21.68 kg (standard deviation 1.04 kg) and an age of approximately 5 months (standard deviation 4 days) were selected for the study. The same scale was used for age standard deviation, i.e., 4 days/30.5 days (1 month) = 0.13 months. All lambs were randomly assigned into two treatments, and received non-Spirulina diet as the control (CK treatment) and the Spirulina added diet (Spirulina was added at a rate of 3% of the fresh weight of the diet). The results indicated that the triacylglycerol (*p* < 0.0001), alanine transaminase (ALT) (*p* < 0.0001), aspartate aminotransferase (AST) (*p* < 0.0001), glucose (*p* < 0.0001), immunoglobulin G (*p* = 0.0066) and insulin (*p* = 0.0025) levels were markedly increased in the Spirulina treatment compared to those in the CK treatment. The principal coordinates analysis showed that the bacterial community did not cluster separately between the CK and Spirulina treatments. Firmicutes, Bacteroidetes and Actinobacteria were the dominant members of the community in two treatments. *Prevotella* were the primary genera, followed by the *Lachnospiraceae*_NK3A20_group, *Olsenella*, *Succinivibrionaceae*_UCG-001 and *Ruminococcus*, and a significant (*p* < 0.05) difference was found in *Olsenella* between the two treatments. These results suggest that the addition of Spirulina is more beneficial for serum biochemical parameters and rumen microbiota of lambs. Overall, these findings contribute to the development of strategies to improve rumen microbial communities for healthy ecosystems on the Mongolian Plateau and provide a scientific basis for the use of Spirulina in feed.

## 1. Introduction

The application of Spirulina (*Arthrospira platensis*) in the field of animal feed has received widespread attention due to its rich nutrient content, easy digestion and absorption. For ruminants (e.g., cattle, sheep, etc.), research on Spirulina as a feed additive has focused on nutritional supplementation and digestibility improvement, immunity and antioxidant enhancement, performance improvement, as well as intestinal health and microbial balance. Studies have shown that Spirulina added to lamb diets increased weight gain rate and feed intake, and improved immune response, blood composition and relative abundance of key cellulolytic species in the rumen, significantly enhancing ruminant health status [1,2,3]. Christodoulou et al. showed that Spirulina supplementation helped to regulate the rumen microbiota of ewes and enhanced ruminant digestive system health [4]. Holman et al. showed that incorporation of Spirulina into ruminant diets not only enhanced animal growth efficiency but also had potential economic benefits [5]. Microalgae, an important and promising alternative resource, are unicellular or multicellular photosynthetic algae found in marine, freshwater and semi-saline environments [6]. These microalgae are widely recognized as important sources of proteins, essential fatty acids, carbohydrates, vitamins, minerals, pigments and antioxidants [6]. A variety of microalgae have been shown to be safe for use in human, animal and poultry feeds [6]. In particular, studies have shown that certain species of microalgae, such as Spirulina and Chlorella, have beneficial effects in poultry feed [7,8,9,10].

Spirulina is a blue-green alga and cultivated for many years because of the valuable nutrient content with enrich protein content, vitamins, minerals and phytopigments [11]. It is considered as a feedstuff with highly remarkably effects to balance the amino acid profile [12]. On the other hand, Spirulina has improved the immune system and increased the animal performance [5]. Previously published research indicated that the external factors, including diet, are markedly associated with the gut microbiomes and serum metabolites [13]. Spirulina has been widely used as an effective alternative dietary source in the poultry diet [14]. The addition of Spirulina to broiler chicken diets could enhance animal performance and nutrient digestibility [15]. Furthermore, the dietary supplementation of Spirulina could also improve the immune status of chickens under chronic heat stress conditions [16]. In summary, Spirulina can positively affect animal health and growth through mechanisms such as immunomodulation, antibacterial and antiviral effects, antioxidant effects, improvement of intestinal health, enhancement of disease resistance and regulation of energy metabolism.

To our knowledge, no studies have been conducted to specifically investigate the effects of Spirulina alone on rumen microbial communities and serum indices in lambs. We hypothesized that Spirulina could induce changes in rumen microbial communities and serum indices in lambs. Therefore, the aim of this study was to determine the specific effects of Spirulina on rumen microbial communities and serum markers in lambs.

## 2. Materials and Methods

### 2.1. Ethical Statement

The animal experiment was approved by the College of Grassland Resources and Environment, Inner Mongolia Agricultural University, and was conducted in strict accordance with the Laboratory Animal Care Regulations.

### 2.2. Animals, Experimental Design and Feeding Management

The feeding trial was conducted between August and November 2020 at the Baotou Green Grass Pasture Development Co. Ltd. (Jiuhuan District, Baotou City, Inner Mongolia Autonomous Region, China). The total experimental period was 75 days, with the first 15 days used to acclimatize the lambs to the new diet and environment, and the next 60 days used for sampling. In the current study a total of thirty-six Hu sheep with an initial body weight of 21.68 ± 1.04 kg and age of 5 months ± 4 days were selected. The same scale was used for age standard deviation, i.e., 4 days/30.5 days (1 month) = 0.13 months. All lambs were randomly grouped into two treatment groups. Each group contained six subgroups, each consisting of three lambs. The experimental diet is listed in Table 1. All lambs were randomly assigned into two treatments, and received a non-Spirulina diet as the control (control treatment, CK) and the Spirulina added diet (Spirulina treatment at 3% of the diet). The feed was offered at 110% of their expected intake. The ration was based on the proportion of fiber in the diet, which was 3:7, and fed twice a day (once at 8 a.m. and once at 5 p.m.), and the experimental lambs were watered ad libitum throughout the experimental period [17]. The samples were dried in a convection oven at 60 °C to 65 °C for 48 h until a constant weight was reached, after which the dry matter (DM) content was calculated. The dried samples were ground using a 1 mm sieve sample mill for further analysis of crude protein (CP), neutral detergent fiber (NDF) and acid detergent fiber (ADF). The analysis of DM and CP followed the standard operating procedures of the AOAC [18], while the determination of ADF and NDF content was based on the method previously published by Van Soest et al. [19].

### 2.3. Serum Parameters Analysis

At the end of the experiment, one lamb from each group was randomly selected for slaughter, giving a total of 12 lambs. Before slaughter, approximately 20 mL of blood was drawn from a vein and collected in vacuum tubes for serum biochemical analysis. These tubes were then cooled on ice and centrifuged at 4 °C for 20 min. The centrifuged plasma was stored at −20 °C until needed for biochemical analysis. Plasma levels of triacylglycerol, urea, insulin, glucose, non-esterified fatty acids (NEFAs), total protein (TP) and total cholesterol were determined using methods similar to those used in Fossati et al. (1982) [20]. Alanine aminotransferase (ALT) and aspartate aminotransferase (AST) activities, as well as high-density lipoprotein cholesterol (HDL-C) and low-density lipoprotein cholesterol (LDL-C) concentrations, were determined by colorimetric methods [21]. Serum levels of albumin, malondialdehyde (MDA), glutathione peroxidase (GSH-Px), superoxide dismutase (SOD), catalase (CAT) and immunoglobulin A (IgA), immunoglobulin G (IgG) and immunoglobulin M (IgM) were determined using assay kits provided by the Nanjing Jianjian Biotechnology Institute (NJIBI).

### 2.4. Rumen Sample Collection, DNA Extraction, 16S rRNA Gene Amplification and Sequencing

After slaughter, approximately 100 mL of ruminal fluid was collected from the lambs and stored in sterilized polypropylene centrifuge tubes. These rumen fluid samples were then rapidly frozen in liquid nitrogen and stored at −80 °C for subsequent analysis. DNA was extracted from rumen samples using a DNA kit (D4015, Omega, Inc., Norwalk, CT, USA) according to the manufacturer’s instructions and the concentration and purity of the extracted DNA was determined using a NanoDrop 2000 UV-vis spectrophotometer (Thermo Scientific, Wilmington, NC, USA). The quality of DNA extracted from rumen samples was assessed by 2% agarose gel electrophoresis. Polymerase chain reaction (PCR) amplification was performed by Majorbio Biomedical Technology Co. Ltd. (Shanghai, China) using primers 338F and 806R to amplify the highly variable V3-V4 region of the bacterial 16S rRNA gene. The paired end sequences were identified and assigned to the appropriate samples based on their unique barcodes, and these barcodes and primer sequences were subsequently removed. FLASH software (version 1.2.8) was utilized to merge the paired end sequences to enhance read length. The fqtrim tool (version 0.94) was applied to quality screen the raw sequences to ensure that high-quality clean data were obtained, and chimeric sequences were excluded using Vsearch software (version 2.3.4). The QIIME2 platform (version 2019.7) was used for sequence import and processing. Sequences were quality controlled and denoised during this process. The processed sequences were clustered by the DADA2 plug-in (version 3.11) to generate a table of amplicon sequence variation (ASV) features to provide a basis for subsequent analyses. Microbial classification was performed using the q2-feature-classifier plugin in the QIIME 2 platform, which is based on scikit-learn’s plain Bayesian classifier. Taxonomic assignment of screened amplicon sequence variants (ASVs) was performed using the SILVA database (version 138) to predict 99% concordance between bacteria and their representative sequences. Species diversity analyses, including alpha diversity (Good’s coverage, Chao1 index and Shannon index) and beta diversity analyses, were performed for each sample under both treatments via the q2-diversity plugin in QIIME2 (version 2019.7). Chao1 was commonly used to estimate unobserved species richness, Shannon’s index was used to measure species diversity, and good’s coverage was used to estimate the completeness of sample coverage. In this study, the weighted UniFrac distance matrix was analyzed using the Principal Coordinate Analysis (PCoA) method to assess and visualize the differences in bacterial communities between individuals and groups. Using the PERMANOVA (version 2.5.4) software package, we determined statistically significant differences in bacterial community composition between the two different treatment conditions [22]. The distribution and structure of the microbial community of rumen samples in each treatment was analyzed using the Majorbio I-Sanger Cloud Platform. Raw sequence data from this study have been submitted to NCBI’s Sequence Read Archive (SRA) under project accession number PRJNA758799.

### 2.5. Statistical Analysis

The serum parameters and microorganism diversity of rumen samples were performed by a one-way analysis of variance with six replicates. Differences in means among the two groups were evaluated using an independent sample *t*-test, and *p* < 0.05 was considered statistically significant [23].

## 3. Results

### 3.1. Analysis of the Diet

The ingredients and chemical composition of the diet used in the current study are shown in Table 1. The DM contents in the CK and Spirulina treatment were 66.69 and 67.03. The CP, ADF and NDF contents in the two treatments were similar from 66.69 to 67.03, 19.11 to 19.56, 14.21 to 19.34 and 24.33 to 24.42, respectively.

### 3.2. Effects of Spirulina on Serum Biochemical Parameters in Lambs

The results of supplementation with Spirulina on serum biochemical parameters of lambs are given in Table 2. Most of the data of the serum biochemical parameters of the lambs were not affected by the treatments, except for the triacylglycerol, ALT, AST and glucose; the other parameters were decreased in the Spirulina treatment compared to the CK treatment. The supplementation with Spirulina markedly (*p* < 0.05) increased the triacylglycerol, ALT, AST and glucose contents compared to the levels in the CK treatment.

### 3.3. Effects of Spirulina on Serum Immunity Statuses in Lambs

The results of supplementation with Spirulina on serum immunity statuses of lambs are given in Table 3. Similarly, with the serum biochemical parameters, the majority of the data of the serum immunity statuses of lambs were not influenced by the treatments. The supplementation with Spirulina markedly increased the IgG (*p* = 0.0066) and insulin (*p* = 0.0025) contents compared to these in the CK treatment, and a significant (*p* < 0.05) difference was observed in the NEFAs (*p* = 0.0055) between the two treatments.

### 3.4. Rumen Microbiota Diversity Analysis

In this study, 12 rumen samples of lambs from CK and Spirulina treatments were collected to investigate the diversity of rumen microbiome by 16S rRNA sequencing. A total of 900,284 and 484,294 sequences and valid sequences from 5383 ASVs, with an average of 40,357 valid sequences (Table 4). The Good’s coverage index for all rumen samples exceeded 0.99, indicating that the sequencing depth was sufficient to comprehensively assess the microbial community in the rumen. The higher α-diversity, including the number of observed ASVs and the Shannon index, was observed in the CK treatment, whereas no significant (*p* > 0.05) differences were found between the CK and Spirulina treatments.

**Figure 1:**
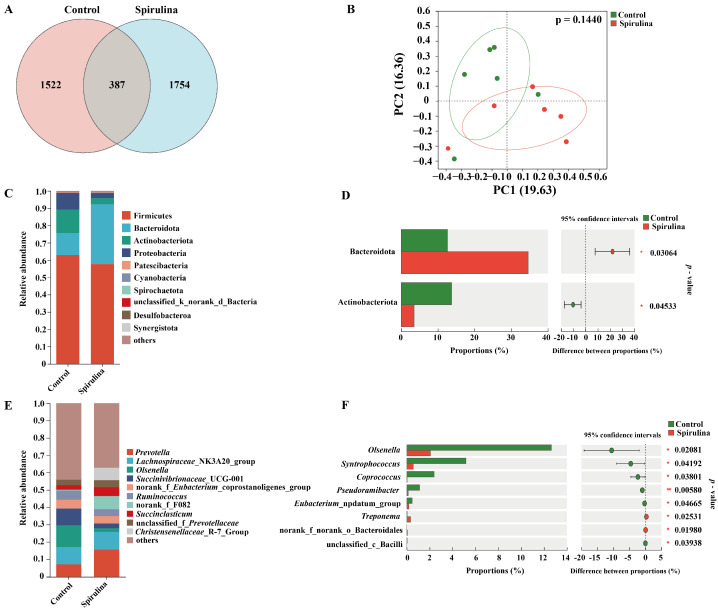
Bacterial community diversities and dissimilarities of rumen samples. (**A**) Venn diagram representing the unique and common ASVs detected at each treatment. (**B**) Principal coordinate analysis (PCoA) of rumen microbial communities based on Bray–Curtis distance. (**C**) The relative abundance (%) of bacterial phyla (1% at least in one group) of ruminal microbiome in two treatments. (**D**) The bar graphs clearly reveal the bacterial species that differed significantly in phylum-level classification between the two treatment conditions. (**E**) The relative abundance (%) of bacterial genera (1% at least in one group) of ruminal microbiome in two treatments. (**F**) The bar graphs clearly reveal the bacterial species that differed significantly in genus-level classification between the two treatment conditions. Control, CK treatment; Spirulina, Spirulina-fed treatment.

The influence of Spirulina on the microbial community are shown in Figure 1. The unique and common ASVs for the CK and Spirulina treatments were different (Figure 1A). The common ASVs were 386, while the unique ASVs for the CK and Spirulina treatments were 1522 and 1754, respectively. The PCoA plot showed that the bacterial community did not cluster separately in the CK and Spirulina treatments (Figure 1B).

At the level of phylum classification, the analysis of all rumen samples identified a total of 14 different phyla, consisting mainly of the Firmicutes, Bacteroidetes, Actinobacteria and Ascomycetes phyla. Of these, the relative abundance of the Firmicutes phylum was the highest, followed by the Bacteroidetes and Actinobacteria phyla, which together accounted for 90% of all sequences (Figure 1C). There was a significant difference in the relative abundance of Bacteroidetes (*p* = 0.03064) and Actinobacteria (*p* = 0.04533) between the two different treatments (Figure 1D). At the genus level, the top 10 genera with the highest relative abundance are shown by stacked bar charts (Figure 1E), with the genus *Prevotella* dominating, followed by *Lachnospiraceae*_NK3A20_group, *Olsenella*, *Succinivibrionaceae*_UCG-001 and *Ruminococcus*.

### 3.5. Mantel Analysis Between the Predominant Rumen Bacteria and Serum Parameters

**Figure 2:**
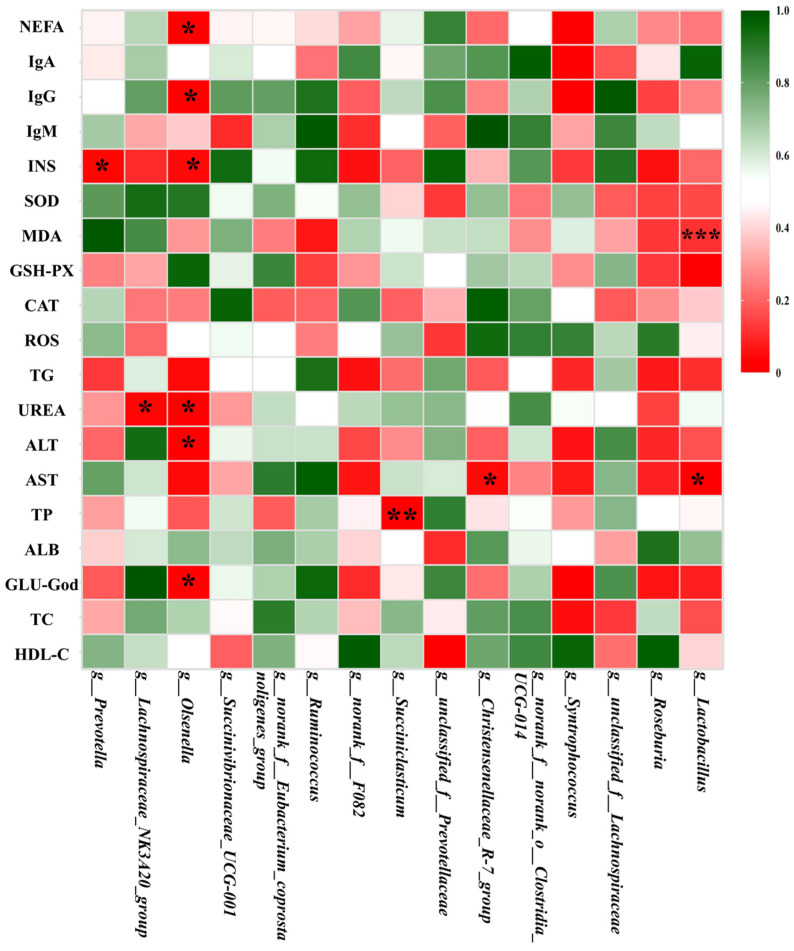
Correlation analysis among the serum parameters and ruminal bacterial community. In the figure, the colors of the grid represent different correlations (red represents a positive correlation and blue represents a negative correlation). * Represents *p* < 0.05, ** represents *p* < 0.01, *** represents *p* < 0.001. Significant differences between the mean values were determined by one-way ANOVA with the LSD test.

To investigate the relationship between serum biochemical indices or immune parameters and changes in the rumen microbial community, a correlation study was performed between the composition of the rumen microbial community and serum biochemical or immune parameters. The results of the analysis (Figure 2) showed a significant correlation between rumen microbial communities and serum biochemical or immune parameters. The genera *Prevotella* and *Lachnospiraceae*_NK3A20_group were positively associated with INS (r = 0.603, *p* < 0.05 and r = −0.855, *p* < 0.05) and UREA (r = 0.601, *p* < 0.05 and r = 0.636, *p* < 0.05), respectively, and *Olsenella* was correlated with the NEFA (r = 0.666, *p* < 0.05), IgG (r = −0.625, *p* < 0.05), INS (r = −0.585, *p* < 0.05), UREA (r = 0.636, *p* < 0.05), ALT (r = −0.668, *p* < 0.05) and Glucose (r = −0.620, *p* < 0.05), respectively. The genus *Lactobacillus* was positively and negatively associated with CAT (0.735, *p* < 0.01) and AST (r = −0.670, *p* < 0.05).

## 4. Discussion

Spirulina contains plenty of bioactive compounds such as vitamins, minerals, antioxidants and γ-linolenic acid, which is a natural feed additive because of the well-known health benefits [3,24]. A previous report indicated that Spirulina could enhance the immune function and antimicrobial activity of the host [25], whereas there is limited information available regarding the effects of Spirulina on the serum parameters and rumen microbiota of lambs. This study integrated blood biochemistry analysis and high-throughput sequencing technology to investigate the effects of Spirulina on serum indicators and rumen microbial communities in lambs, laying the foundation for further research in this area.

The higher levels of triacylglycerol, total protein and albumin and lower cholesterol were observed for Spirulina treatment, which was similar to previous reports that the addition of Spirulina increases total protein and albumin and decreases cholesterol [25,26,27] due to the fact that the addition of Spirulina reduces the level of lipids [28]. In this current study, the serum biochemical parameters were increased in the Spirulina treatment, especially the ALT and AST parameters, which could be explained by the abundant proteins, polysaccharides, carotenoids and other bioactive compounds with antioxidant action [3,29]. The level of blood glucose is considered as a marker to determine stress and an adaption to meet the demand [30]. The bioactive compounds or polyphenols in Spirulina can promote health by inactivating α-amylase and α-glucosidase [31,32], which leads to the higher glucose levels in the Spirulina treatment. Hyperlipidemia is one of the most common lipid metabolism disorders and illustrated by low HDL-C and high LDL-C [33]. In the current study, the higher and lower HDL-C and LDL-C levels were observed in the Spirulina treatment. The rich amino acid composition of Spirulina may affect lipid metabolism through a variety of mechanisms, including direct involvement in the regulation of cholesterol synthase activity, participation in lipoprotein synthesis and synergistic effects with other active ingredients, which may increase HDL-C levels and decrease LDL-C levels [33,34].

As markers of the immune system, IgA, IgG and IgM are the key factors to reflect the immune function in the serum of animals [35]. The higher content of hippuric acid produced by phenylalanine and tyrosine metabolism might lead to the higher levels of IgA, IgG and IgM in the Spirulina treatment [36]. Due to the antioxidant content of Spirulina, more and more studies are beginning to explore the possibility of using it as a feed additive to mitigate oxidative damage [11]. Key antioxidant enzymes such as superoxide dismutase (SOD), malondialdehyde (MDA), glutathione peroxidase (GSH-Px) and catalase (CAT) are often used as key indicators of oxidative stress levels [37,38]. An increase in the activity of these antioxidant enzymes was observed in the Spirulina-treated group, which had a positive effect on enhancing the immune defenses of the lambs. These results are consistent with previous findings suggesting that Spirulina has significant antioxidant capacity and may have a beneficial effect on animal health [39].

The role of Spirulina may be to modulate the metabolic activities of the gut microbiota rather than directly altering the structure of the microbial community. For example, Spirulina polysaccharides increased the number of some beneficial bacteria such as *Lactobacillus*, *Prevotella* and *Olsonella*, while decreasing the number of some conditionally pathogenic bacteria [40]. This could be the reason why there was no significant difference (*p* > 0.05) in the number of ASVs, Chao1 value and Shannon index between the two treatments. These results could be explained by the Spirulina having a positive effect to kill microbes by increasing the defense system [41]. All samples achieved a Good’s coverage of 0.99 or higher, a figure indicating that the depth of sequencing performed was sufficient to accurately represent the bacterial community in the rumen samples [42]. To investigate the potential effect of Spirulina on beta diversity, we analyzed the rumen microbial community based on weighted UniFrac distances of all rumen samples. Using PCoA mapping, we observed significant differences in the bacterial communities between treatment groups, which is consistent with previous findings that different feed types can lead to changes in rumen pH, which in turn affects the microbial composition within the rumen [40].

In the present study, we found that Firmicutes and Actinomycetes were the dominant phyla in the rumen, which is consistent with previous findings that these two phyla dominate the rumen microbial community of sheep [40]. Compared to the control (CK) group, the Spirulina-treated group showed a significant increase in the number of taxonomic phyla (*p* = 0.03064) and a decrease in the number of Actinomycetes (*p* = 0.04533), which is consistent with previous findings [43]. At the genus level, the most abundant bacteria in the rumen included *Prevotella*, *Lachnospiraceae*_NK3A20_group, *Olsenella* and *Succinivbrionaceae*_UCG-001. The genus *Prevotella* plays a key role in fiber attachment and degradation as it is able to break down fibrous material [44]. *Prevotella* spp. were also the most abundant genera in the rumen of ruminants, which is in line with previous findings [45,46]. There was an increase in the number of *Prevotella* spp. in the Spirulina-treated group, which may be related to the addition of Spirulina. A significant (*p* < 0.05) difference was found between the two treatments and the markedly lower *Olsenella* was observed in the Spirulina treatment, which could be explained by the addition of polysaccharide content in the Spirulina diet [47]. In this study, Mantel analysis was used to investigate potential associations between serum parameters and major genera of rumen microbiota in lambs. The results showed that *Lactobacillus* spp. were positively correlated with GSH-Px and negatively correlated with AST. This correlation may be related to the role of *Lactobacillus* probiotics in regulating disorders of lipid and carbohydrate metabolism, and they may exert their probiotic effects by influencing metabolic pathways in the host [47,48].

## 5. Conclusions

The present study demonstrated that the Spirulina had a positive effect on the rumen microbiome and serum parameters. Triacylglycerol, ALT, AST and Glucose contents were significantly increased by the addition of 3% Spirulina to the diet of Hu sheep, and regulated the intestinal microbiota and increases the abundance of beneficial bacteria (*Prevotella*, *Lachnospiraceae*_NK3A20_group, *Olsenella* and *Succinivbrionaceae*_UCG-001). These findings will help develop strategies to improve rumen microbial communities in the Mongolian Plateau ecosystem, which in turn will improve the health of the entire ecosystem, and provide a scientific basis for the use of Spirulina in the diet.

## Figures and Tables

**Table 1:** Ingredients and chemical composition of the diets.

Item	CK	Spirulina
Ingredients (%)		
Maize	42.63	42.63
Soybean meal	19.60	19.60
Flax	7.84	7.84
Alfalfa	16.10	15.80
Wheat bran	5.88	5.88
Premix	5.00	5.00
Salt	0.98	0.98
Soda	1.17	1.17
Hawthorn	0.10	0.10
Malt	0.10	0.10
Dried tangerine peel	0.10	0.10
Medicated Leaven	0.10	0.10
Astragalus membranaceus	0.10	0.10
Atractylodes macrocephala	0.10	0.10
Licorice	0.10	0.10
Epimedium	0.10	0.10
Spirulina	0.00	0.30
Chemical compositions		
Dry matter (%)	66.69	67.03
Crude protein (%DM)	19.56	19.11
Acid detergent fiber (%DM)	14.21	14.34
Neutral detergent fiber (%DM)	24.42	24.33

CK, Control treatment.

**Table 2:** Effect of feed supplementation with Spirulina on serum biochemical parameters of lambs.

Item	CK	Spirulina	*p*-Value
Triacylglycerol (mmol/L)	0.16 ± 0.01 b	0.41 ± 0.02 a	<0.0001
Urea (mmol/L)	3.02 ± 0.12	2.97 ± 0.14	0.8516
ALT (U/L)	11.35 ± 1.16 b	40.98 ± 1.50 a	<0.0001
AST (U/L)	74.63 ± 2.07 b	191.73 ± 17.33 a	<0.0001
Total protein (g/L)	65.88 ± 2.30	66.58 ± 1.26	0.7950
Albumin (g/L)	22.60 ± 0.75	21.00 ± 0.43	0.0928
Glucose (mmol/L)	5.77 ± 0.20 b	13.40 ± 0.33 a	<0.0001
Total cholesterol (mmol/L)	1.34 ± 0.08	1.14 ± 0.05	0.0657
HDL-C (mmol/L)	0.44 ± 0.09	0.42 ± 0.02	0.8487
LDL-C (mmol/L)	0.40 ± 0.04	0.33 ± 0.02	0.1217

ALT, alanine aminotransaminase; AST, aspartate aminotransferase; CK, Control treatment; HDL-C, high-density lipoprotein cholesterol; LDL-C, low-density lipoprotein cholesterol. Different lowercase letters indicate significant differences between treatments (*p* < 0.05).

**Table 3:** Effect of feed supplementation with Spirulina on serum immunity statuses of lambs.

Item	CK	Spirulina	*p*-Value
NEFAs (mmol/L)	0.65 ± 0.05 a	0.45 ± 0.03 b	0.0055
IgA (g/L)	0.35 ± 0.02	0.34 ± 0.01	0.5625
IgG (g/L)	8.30 ± 0.34 b	9.73 ± 0.24 a	0.0066
IgM (g/L)	0.57 ± 0.02	0.59 ± 0.02	0.5219
Insulin (uIU/mL)	11.13 ± 0.26 b	13.28 ± 0.47 a	0.0025
SOD (U/mL)	79.22 ± 01.50	79.43 ± 1.14	0.9136
MDA (nmol/mL)	2.66 ± 0.21	3.23 ± 0.27	0.1308
GSH-Px (U/mL)	176.28 ± 5.70	178.92 ± 5.10	0.8621
CAT (U/mL)	40.17 ± 1.32	43.87 ± 0.84	0.1731

CK, Control treatment; NEFAs, non-esterified fatty acids; IgA, immunoglobulin A; IgG, immunoglobulin G; IgM, immunoglobulin M; SOD, superoxide dismutase; MDA, malondialdehyde; GSH-Px, glutathione peroxidase; CAT, Catalase. Different lowercase letters indicate significant differences between treatments (*p* < 0.05).

**Table 4:** Effects of feed supplementation with Spirulina on diversity indices of ruminal microbiome of lambs.

Item	CK	Spirulina	*p*-Value	Total No.
No. of sequences	73,420 ± 3169	76,627 ± 4896	0.5939	900,284
No. of valid sequences	40,392 ± 2385	40,323 ± 1596	0.9813	484,294
Observed ASV number	468 ± 70	428 ± 69	0.6925	
Good’s coverage	>0.99	>0.99	0.9221	
Chao1 value	470.98 ± 71.12	429.81 ± 70.61	0.6899	
Shannon index	4.03 ± 0.22	3.08 ± 0.29	0.6838	

CK, Control treatment.

## Data Availability

The original contributions presented in the study are included in the article, further inquiries can be directed to the corresponding author.
